# Polygenic risk for schizophrenia and subcortical brain anatomy in the UK Biobank cohort

**DOI:** 10.1038/s41398-020-00940-0

**Published:** 2020-09-09

**Authors:** Steluta Grama, Isabella Willcocks, John J. Hubert, Antonio F. Pardiñas, Sophie E. Legge, Matthew Bracher-Smith, Georgina E. Menzies, Lynsey S. Hall, Andrew J. Pocklington, Richard J. L. Anney, Nicholas J. Bray, Valentina Escott-Price, Xavier Caseras

**Affiliations:** 1 MRC Centre for Neuropsychiatric Genetics and Genomics, Division of Psychiatry and Clinical Neurosciences, Cardiff University, Cardiff, CF10 3AT UK; 2 UK Dementia Research Institute, Cardiff University, Cardiff, CF10 3AT UK

**Keywords:** Predictive markers, Schizophrenia

## Abstract

Research has shown differences in subcortical brain volumes between participants with schizophrenia and healthy controls. However, none of these differences have been found to associate with schizophrenia polygenic risk. Here, in a large sample (*n* = 14,701) of unaffected participants from the UK Biobank, we test whether schizophrenia polygenic risk scores (PRS) limited to specific gene-sets predict subcortical brain volumes. We compare associations with schizophrenia PRS at the whole genome level (‘genomic’, including all SNPs associated with the disorder at a *p*-value threshold < 0.05) with ‘genic’ PRS (based on SNPs in the vicinity of known genes), ‘intergenic’ PRS (based on the remaining SNPs), and genic PRS limited to SNPs within 7 gene-sets previously found to be enriched for genetic association with schizophrenia (‘abnormal behaviour,’ ‘abnormal long-term potentiation,’ ‘abnormal nervous system electrophysiology,’ ‘FMRP targets,’ ‘5HT2C channels,’ ‘CaV2 channels’ and ‘loss-of-function intolerant genes’). We observe a negative association between the ‘abnormal behaviour’ gene-set PRS and volume of the right thalamus that survived correction for multiple testing (ß = −0.031, p_FDR_ = 0.005) and was robust to different schizophrenia PRS *p*-value thresholds. In contrast, the only association with genomic PRS surviving correction for multiple testing was for right pallidum, which was observed using a schizophrenia PRS *p*-value threshold < 0.01 (ß = −0.032, *p* = 0.0003, p_FDR_ = 0.02), but not when using other PRS *P*-value thresholds. We conclude that schizophrenia PRS limited to functional gene sets may provide a better means of capturing differences in subcortical brain volume than whole genome PRS approaches.

## Introduction

Schizophrenia (SZ) is a highly heritable (h^2^ ~ 80%)^1^ psychiatric disorder that can carry a severe burden for affected individuals and their families. Recent advances in our understanding of the genetic risk for SZ have confirmed its polygenic nature, with hundreds—if not thousands^2^—of common risk alleles explaining ~25% of the variance in liability to the disorder^3^. The most recent published Genome Wide Association Study (GWAS) identified 145 independent loci associated with SZ at genome-wide significance^4^. Despite the increase in our understanding of the genetic factors related to SZ, our knowledge about its neurobiological underpinnings remains scarce. Although recent large multicentric studies have consistently shown differences in subcortical brain volumes between SZ participants and healthy controls^5,6^, research looking at the combined risk effect of common alleles using polygenic risk scores (PRS)^7^ has shown no association between PRS for SZ and subcortical volumes^8–10^. This could suggest that these differences in subcortical volumes are a consequence, as opposed to a premorbid risk marker, of schizophrenia; albeit other interpretations are also plausible, for example that these are driven by environmental risk. However, previous research has also shown similar subcortical abnormalities in unaffected relatives of SZ patients^11–14^ and in healthy carriers of rare alleles penetrant for SZ^15^, suggesting that at least some of these subcortical differences could well represent intermediate phenotypes for SZ that lie on the causal pathway between genetic variation and expression of the disorder. Identifying brain intermediate phenotypes is key for our understanding of the mechanisms behind SZ, unveiling its etiopathogenesis and ultimately developing more effective clinical interventions.

An alternative explanation for the lack of association could be the potential heterogeneity in the effect of risk alleles across the brain. It is possible that only a subset of SZ associated variants would relate to the size of subcortical structures^16^, and even then not all subcortical volumes would necessarily associate to the same subset of variants. So far, research in this area has applied PRS optimised to account for the presence of SZ in the population, but this should not necessarily be optimal to account for brain variability. PRS based on biologically driven gene-sets may outperform the genome-wide PRS in explaining brain phenotypes. Examples of that are recent studies attempting to identify ‘core gene-sets’ that make a larger contribution to SZ risk^17^, or to predict brain anatomy^18^ and functional connectivity^19^ via PRS limited to genes up- or down-regulated by MIR137^20^.

In this proof-of-concept exploratory study we aim to revisit the association between common allele risk for SZ and subcortical brain volumes using different strategies to calculate PRS in a large sample of unaffected participants from the UK Biobank cohort. We compare the associations of the genome-wide PRS for SZ (henceforth *genomic PRS*) with subcortical brain volumes, to those associations where PRS is limited to loci within genes (*genic PRS*) or loci outside those genomic regions (*intergenic PRS*). We also calculate PRS based on seven gene sets that previous research has shown to be particularly enriched for common and rare SZ risk variants^4,21^. These will serve the purpose of testing our hypothesis that reducing heterogeneity within the SNPs included in PRS improves its ability to predict subcortical volume variability, guiding future research in this area towards identifying gene-sets that maximise brain phenotype variance explained, rather than simply adding new significant SNPs from future GWASs through an ‘omic’ PRS approach. However, we are assuming here that these gene sets have the ability to influence brain structure, and it should also be noted that other gene-sets based on different criteria—e.g., gene ontology terms—could have been equally selected. We are also hypothesising that different gene-set PRS will show association with particular subcortical volumes, showing heterogeneity of effects across them, rather than suggesting a common mechanism of action. We expect PRS to show the same direction of effect shown in clinical research: negative associations between the volume of most subcortical structures and PRS, but positive for pallidum and the putamen.

## Methods

### Participants

This study used a subsample of participants from the UK Biobank (www.ukbiobank.ac.uk), a UK population cohort of ~500,000 participants recruited between 2006 and 2010 when aged 40–69 years, and followed-up since. Our initial sample included those participants from whom brain MRI anatomical images (T1) were available at the time of the analyses (*n* = 20,664). All subjects provided informed consent to participate in UK Biobank. Ethical approval was granted by the North West Multi-Centre Ethics committee. Data were released to Cardiff University after application project reference 17044.

Only participants self-reporting White British or Irish descent for whom European ancestry was subsequently confirmed through genetic analysis^22^ were included in the study. Related participants up to second degree relatives (kinship coefficient >0.15) were excluded by randomly removing one participant from the related pair. Furthermore, in order to avoid reverse causation effects, participants were excluded if a personal history for severe psychiatric disorders (i.e., SZ, psychosis, bipolar disorder, autistic spectrum disorder or intellectual disability) or medical/neurological conditions that could affect brain anatomy (i.e., alcohol dependence, dementia, Parkinson’s, neurodegenerative disorders or brain cancer) were recorded through self-reported diagnosis from a doctor, hospital records, or death records.

After applying the above inclusion/exclusion criteria and the genetic QC below, the final sample included 14,701 participants, with a mean age of 62 years (sd: 7.4, range: 45–80) and 51% female.

### Genotyping and PRS computation

Genotyping was performed by UK Biobank using the Affymetrix UK BiLEVE Axiom array (807,411 probes) on an initial 50,000 participants, and the Affymetrix UK Biobank Axiom® array (820,967 probes) for the remaining participants. The two arrays are extremely similar (with over 95% common content). Sample processing at UK Biobank has been previously described^23^ and resulted in the release of the 488,377 samples that went into our PRS calculations.

In order to calculate PRS, a random subset of 5000 UK biobank participants not included here and matched by age and gender distribution with our sample were used as an LD reference dataset for pruning and clumping of SNPs (7,232,075 total). We retained SNPs in our sample with a minor allele frequency ≥5% and a Hardy–Weinberg equilibrium *p*-value ≥ 10^−6^. Correlated SNPs were pruned using *r*^2^ = 0.2, a physical distance threshold of 1 Mb, preferentially retaining the SNP most significantly associated with SZ^4^. SNPs within the extended MHC locus (chr6:25 Mb–35 Mb) were excluded due to high levels of linkage disequilibrium in the region, as were insertion/deletion polymorphisms and ambiguous flip SNPs. Genetic principal components 1–10 were included as population stratification covariates in the calculation of the scores. We initially used a *p*-threshold of 0.05 since this has shown to maximally capture polygenic risk across a large number of independent samples^24^. For comparison, analyses spanning a range of *p*-thresholds (0.5, 0.1, 0.01, 0.001, 0.0001) were also conducted. All scores were calculated in PLINK v1.90b5.4 64-bit via Stata/IC 13.1 (https://github.com/ricanney/stata) *using best-guess genotypes.*

For *genomic PRS*, full summary statistics from the latest available GWAS^4^ were used (7,248,434 SNPs). For *genic PRS*, the summary file was trimmed to contain only SNPs that were located within 35 Kb upstream and 10 Kb downstream of genes detailed in the comprehensive GENCODE 31 human gene list (https://www.gencodegenes.org). Pseudogenes were removed, leaving a total of 19,965 genes, which produced a summary file containing 5,363,146 SNPs. The *intergenic PRS* was calculated from the SNPs that were not retained in the *genic PRS* summary file (1,885,288 SNPs). Seven gene-set PRS were generated following the exact same steps than used for the *genic PRS*, but in this case SNPs included in each PRS were limited to those contained in genes within that gene set. Three of these sets derive from the Mouse Genome Informatics database^25^ and relate to behavioural and neurophysiological correlates of learning: *abnormal behaviour* (MP:0004924; 717,522 SNPs spanning over 2037 genes included in the PRS), *abnormal long term potentiation* (MP:0002207; 68,686 SNPs from 157 genes included in the PRS), *abnormal nervous system electrophysiology* (MP:0002272; 106,641 SNPs included in 213 genes considered in the PRS). The other four sets included: targets of the fragile X mental retardation protein^26^ *(FMRP targets*; 403,723 SNPs from 839 genes included in the PRS), the 5-HT_2C_ receptor complex^27^ *(5HT2C channels*; 4435 SNPs included in 18 genes considered in the PRS), the voltage-gated calcium channel complexes^28^ (*CaV2 channels*;107,987 SNPs from 207 genes included in the PRS) and loss of function intolerant genes as defined by the Exome Aggregation Consortium^29^ using their gene-level constraint metric (pLI ≥ 0.9) (*LoF intolerant*; 1,152,144 SNPs from 3,191 genes included in the PRS).

### Subcortical volumes

Brain images were acquired using Siemens Skyra 3T scanners in UK Biobank’s imaging centres in Cheadle (*n* = 7683), Reading (*n* = 5599) and Newcastle (*n* = 1419) using identical acquisition protocols (http://biobank.ctsu.ox.ac.uk/crystal/refer.cgi?id=2367). T1-weighted NIfTI brain images were processed in our lab using FreeSurfer v5.3 (https://surfer.nmr.mgh.harvard.edu) to automatically obtain estimates of subcortical volumes (i.e., thalamus, caudate, putamen, pallidum, nucleus accumbens, hippocampus and amygdala). To avoid values potentially resulting from deficient segmentation of tissue types, extreme values defined as ±3 standard deviations from the group mean were removed from each corresponding analysis (0.3–0.8% of the total sample). Bilateral subcortical volumes were obtained by averaging the volume of left and right subcortical structures.

### Analyses

Regression analyses were run with each bilateral brain volume as the outcome variable and PRS as predictor, after accounting for the effect of intracranial volume, sex, age and acquisition centre, using IBM SPSS Statistics v25. Although we accounted for population stratification when calculating PRS, the first 5 genetic principal components were also included as covariates in the regression models to control for any potential residual effects. Significance threshold was set at *p* < 0.05 (two-sided), and False Discovery Rate (FDR; 0.1) based on Benjamini–Hochberg^30^ was used to correct for multiple testing. Due to the exploratory nature of this study, we report uncorrected (nominal) *p* values throughout, along with the corrected *p* value (p_FDR_) for those associations that survive multiple testing correction. We first tested the association between *genomic PRS* and the volume of each seven subcortical structures; followed by the same analyses for *genic PRS* and *intergenic PRS*. Finally, we tested the association of each gene-set PRS against each subcortical volume. In order to examine potential interhemispheric differences, further analyses were run for each hemisphere. Finally, to explore the optimal PRS *p*-threshold to inform future research and to examine the stability of results as an index of validity, we expanded our analyses to PRS calculated under a wider range of *p*-thresholds (i.e., 0.5, 0.1, 0.01, 0.001, 0.0001). We compared the performance of different PRS based on the statistical significance of the associations found, their effect size (i.e., beta value) and their stability across different *p*-thresholds. Therefore, we considered the best PRS predictive of subcortical volumes to be those that produced FDR-corrected significant associations with the highest beta value, reproduceable at different *p*-thresholds.

## Results

### PRS (*p*-threshold < 0.05) associations with left-right averaged subcortical volumes

No association was found between *genomic PRS* and subcortical volumes. Likewise, *genic PRS* did not show association with any subcortical volumes. *Intergenic PRS*, though, showed *negative* nominal significant associations with the size of the putamen and the amygdala, although neither of these were significant after FDR correction (Table 1).

**Table 1:** Association (Beta) between polygenic scores (genomic, genic, intergenic and gene-sets) and subcortical volumes averaged across hemispheres.

	Thalamus	Caudate	Putamen	Pallidum	Accumbens	Hippocampus	Amygdala
PRS _Pt=0.05_	β (*p*)	β (*p*)	β (*p*)	β (*p*)	β (*p*)	β (*p*)	β (*p*)
Genomic	−0.006 (0.41)	−0.015 (0.10)	−0.012 (0.19)	−0.013 (0.17)	−0.006 (0.51)	−0.007 (0.45)	−0.008 (0.34)
Genic	−0.008 (0.26)	−0.013 (0.13)	−0.012 (0.19)	−0.010 (0.29)	−0.007 (0.47)	−0.007 (0.44)	−0.006 (0.49)
Intergenic	−0.001 (0.87)	−0.014 (0.15)	−*0.018* (*0*.*05)*	−0.016 (0.09)	−0.011 (0.25)	−0.008 (0.37)	−*0.021* (*0*.*02)*
5HT2C	0.004 (0.63)	−0.003 (0.75)	−0.005 (0.57)	−0.006 (0.54)	−0.007 (0.46)	−0.002 (0.82)	0.007 (0.43)
abnormal_beh	−*0.030* (*0*.00005)*	−0.014 (0.10)	−0.004 (0.68)	0.003 (0.75)	−0.003 (0.73)	−0.013 (0.12)	−0.001 (0.87)
abnormal_ltp	−0.007 (0.32)	−0.016 (0.07)	−0.007 (0.42)	0.01 (0.29)	−0.002 (0.83)	−0.001 (0.88)	0.005 (0.55)
abnormal_nse	0.010 (0.19)	−0.014 (0.10)	−0.012 (0.18)	−0.003 (0.75)	−0.004 (0.69)	0.006 (0.50)	0.001 (0.95)
CaV2 channels	0.002 (0.78)	−0.001 (0.90)	−0.000 (0.97)	0.008 (0.41)	−0.009 (0.35)	0.004 (0.64)	0.003 (0.75)
FMRP targets	−*0.019* (*0*.*01)*	−0.010 (0.27)	0.011 (0.21)	*0.019* (*0*.*04)*	−0.004 (0.66)	−*0.021* (*0*.*01)*	−0.002 (0.82)
LoF intolerant	−*0.016* (*0*.*03)*	0.002 (0.82)	−0.002 (0.84)	0.002 (0.82)	0.011 (0.26)	−0.011 (0.19)	−0.002 (0.85)

*P* values reported in this table are uncorrected. Nominally significant associations are highlighted in italic. Significant associations after FDR correction (7 tests for genomic PRS, 14 for genic/intergenic PRS, 49 for gene−set PRS) are highlighted with an asterisk. 5HT2C: 5−HT_2C_ receptor complex genes set; abnormal_beh: abnormal behaviour genes set; abnormal_ltp: abnormal long term potentiation genes set; abnormal_nse: abnormal nervous system electrophysiology; CaV2 channels: voltage-gated calcium channel complexes genes set; FMRP targets: targets of fragile X mental retardation protein genes set; LoF intolerant: loss of function intolerant genes set.

Three *gene set PRS—abnormal behaviour*, *FMRP targets* and *LoF intolerant*—showed negative associations with the volume of the thalamus, although only the former remained significant after FDR correction (β = −0.030, *p* = 0.00005, p_FDR_ = 0.002, adjusted *R*^2^ = 0.50, *R*^2^ change = 0.001). *FMRP targets* gene-set PRS was also associated positively with the volume of the pallidum and negatively with the hippocampus, although only at nominal level (Table 1).

### Within-hemisphere analyses

*Genomic PRS* showed negative nominal associations with left caudate and putamen, and right pallidum; none of these significant after FDR correction. The same nominal associations were found for the *genic PRS*; whereas, again, *intergenic PRS* appeared more strongly associated—negative—with the volume of the amygdala in the right hemisphere (Table 2).

**Table 2:** Association between polygenic scores (genomic, genic, intergenic and gene−sets) and subcortical volumes for left and right hemispheres separately.

	Thalamus	Caudate	Putamen	Pallidum	Accumbens	Hippocampus	Amygdala
PRS _Pt=0.05_	β (*p*)	β (*p*)	β (*p*)	β (*p*)	β (*p*)	β (*p*)	β (*p*)
Left hemisphere							
Genomic	−0.005 (0.52)	−*0.017* (*0*.*05)*	−*0.019* (*0*.*04)*	−0.008 (0.43)	−0.009 (0.37)	−0.014 (0.12)	−0.008 (0.39)
Genic	−0.004 (0.65)	−*0.018* (*0*.*05)*	−*0.020* (*0*.*03)*	−0.006 (0.53)	−0.009 (0.34)	−0.010 (0.26)	−0.005 (0.60)
Intergenic	−0.004 (0.64)	−0.014 (0.12)	−*0.019* (*0*.*04)*	−0.011 (0.28)	−0.014 (0.16)	−0.010 (0.28)	−0.012 (0.18)
5HT2C	0.010 (0.23)	−0.000 (0.96)	−0.007 (0.44)	−0.004 (0.65)	−0.011 (0.23)	−0.002 (0.86)	0.001 (0.91)
abnormal_beh	−*0.020* (*0*.*01)*	−0.013 (0.15)	−0.010 (0.26)	0.002 (0.81)	−0.010 (0.27)	−0.011 (0.19)	−0.012 (0.16)
abnormal_ltp	−0.008 (0.33)	−0.013 (0.14)	−0.008 (0.36)	0.006 (0.55)	0.003 (0.73)	0.004 (0.68)	−0.002 (0.82)
abnormal_nse	−0.004 (0.58)	−0.017 (0.06)	−0.013 (0.15)	−0.003 (0.74)	−0.001 (0.88)	0.004 (0.61)	−0.011 (0.22)
CaV2 channels	0.005 (0.54)	−0.005 (0.61)	−0.002 (0.82)	0.005 (0.63)	−0.003 (0.75)	−0.003 (0.73)	−0.000 (0.98)
FMRP targets	−*0.017* (*0*.*03)*	0.004 (0.63)	0.005 (0.59)	0.017 (0.09)	−0.005 (0.57)	−0.015 (0.08)	−0.004 (0.64)
LoF intolerant	−0.010 (0.21)	−0.001 (0.90)	−0.008 (0.38)	−0.000 (0.97)	0.011 (0.24)	−0.007 (0.39)	−0.003 (0.76)
Right hemisphere							
Genomic	−0.008 (0.30)	−0.013 (0.14)	−0.002 (0.86)	−*0.022* (*0*.*02)*	−0.009 (0.35)	−0.010 (0.25)	−0.013 (0.12)
Genic	−0.009 (0.21)	−0.014 (0.11)	−0.003 (0.70)	−*0.018* (*0*.*04)*	−0.003 (0.74)	−0.006 (0.48)	−0.005 (0.60)
Intergenic	−0.001 (0.88)	−0.013 (0.16)	−0.010 (0.25)	−0.015 (0.09)	−0.015 (0.12)	−0.010 (0.27)	−*0.023* (*0*.*009)*
5HT2C	0.006 (0.41)	−0.002 (0.80)	−0.004 (0.65)	−0.000 (0.98)	−0.008 (0.41)	−0.002 (0.82)	0.010 (0.24)
abnormal_beh	−*0.031* (*0*.00005)*	−0.008 (0.34)	0.004 (0.65)	−0.000 (0.96)	0.004 (0.70)	−0.014 (0.11)	0.010 (0.26)
abnormal_ltp	−0.009 (0.26)	−*0.018* (*0*.*04)*	*−0.018* (*0*.*04)*	0.001 (0.93)	−0.003 (0.73)	−0.002 (0.80)	0.011 (0.22)
abnormal_nse	−0.010 (0.19)	−0.008 (0.35)	−0.005 (0.59)	0.002 (0.84)	−0.005 (0.56)	0.003 (0.75)	*0.017* (*0*.*05)*
CaV2 channels	−0.004 (0.56)	0.004 (0.68)	0.009 (0.32)	0.009 (0.32)	−0.009 (0.35)	−0.001 (0.95)	−0.000 (0.96)
FMRP targets	−0.015 (0.06)	0.009 (0.29)	0.004 (0.64)	0.008 (0.38)	−0.001 (0.89)	−0.016 (0.06)	−0.005 (0.56)
LoF intolerant	−0.010 (0.19)	0.009 (0.29)	0.006 (0.50)	−0.006 (0.53)	0.015 (0.12)	−0.011 (0.21)	0.001 (0.91)

*P* values reported in this table are uncorrected. Nominally significant associations are highlighted in italic. Significant associations after FDR correction (14 tests for genomic PRS, 28 for genic/intergenic PRS, 98 for gene-set PRS) are highlighted with an asterisk. 5HT2C: 5-HT_2C_ receptor complex genes set; abnormal_beh: abnormal behaviour genes set; abnormal_ltp: abnormal long term potentiation genes set; abnormal_nse: abnormal nervous system electrophysiology; CaV2 channels: voltage-gated calcium channel complexes genes set; FMRP targets: targets of fragile X mental retardation protein genes set; LoF intolerant: loss of function intolerant genes set.

G*ene set PRS* analyses showed similar results than above, although the negative association between *abnormal behaviour PRS* and thalamic volume was now only FDR-corrected significant for the right hemisphere (β = −0.031, *p* = 0.00005, p_FDR_ = 0.005, adjusted *R*^2^ = 0.46, *R*^2^ change = 0.001; Table 2).

### Alternative *p*-thresholds

**Fig. 1:**
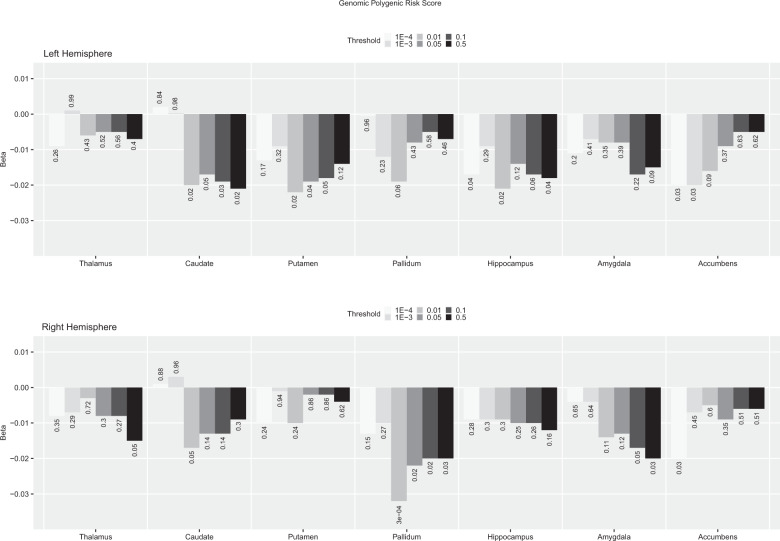
*Genomic* PRS—calculated at different *p* thresholds—association with left and right subcortical volumes. The direction and length of the bar correspond to the Beta value, the associated uncorrected *p* value is added for each Beta/bar. For comparison, this figure also includes the results for the *p*-threshold < 0.05 used in previous analyses. FDR correction based on 70 tests (FDR significant results are mark with an asterisk).

**Fig. 2:**
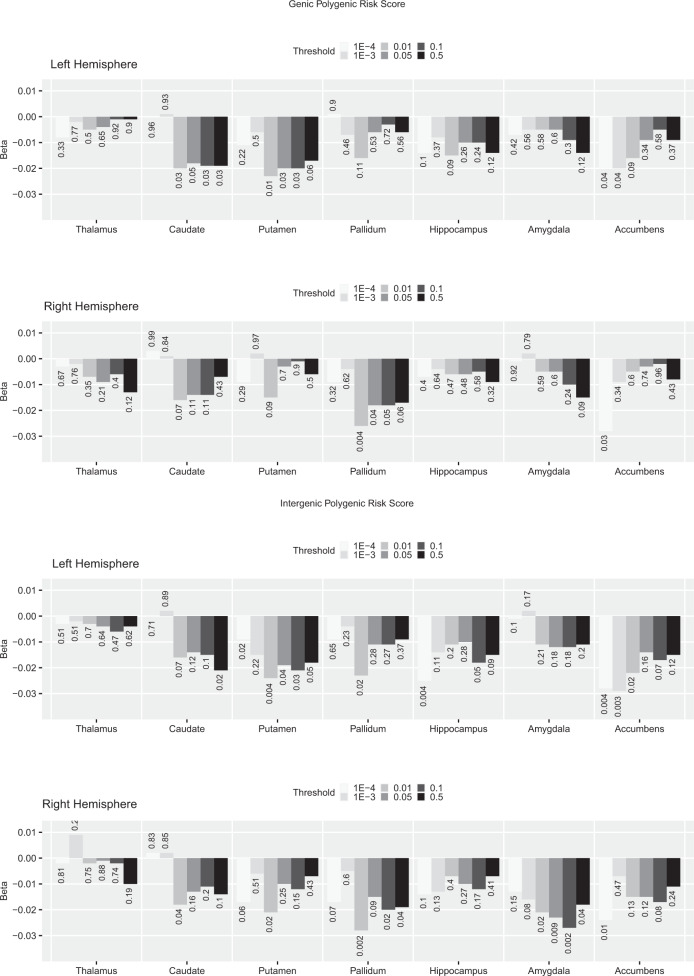
*Genic* (top two) and *intergenic* (bottom two) PRS—at different *p*-thresholds—association with left and right subcortical volumes. The direction and length of the bar correspond to the Beta value, the associated uncorrected *p* value is added for each Beta/bar. For comparison, this figure also includes the results for the *p*-threshold < 0.05 used in previous analyses. FDR correction based on 70 tests.

*Genomic PRS* showed negative nominal associations with the volume of the right pallidum at several *p*-thresholds, this surviving FDR-correction at *p*-threshold <0.01 (ß = −0.032, *p* = 0.0003, p_FDR_ = 0.02, adjusted *R*^2^ = 0.27, *R*^2^ change = 0.0003; Fig. 1). A similar pattern of associations was found for *genic PRS*, again with the strongest association shown at *p*-threshold 0.01 with the right pallidum, although in this case it did not survive FDR correction. *Intergenic PRS* showed overall a similar pattern of associations to *genomic* and *genic* PRS, but in relation to the right amygdala. In this case, *intergenic PRS* showed a negative nominal association across most *p*-thresholds that was not present in *genic PRS*, with the largest effect size shown at *p*-threshold 0.1 (β = −0.027, *p* = 0.002, p_FDR_ = 0.14, adjusted *R*^2^ = 0.32, *R*^2^ change = 0.001; Fig. 2).

**Fig. 3:**
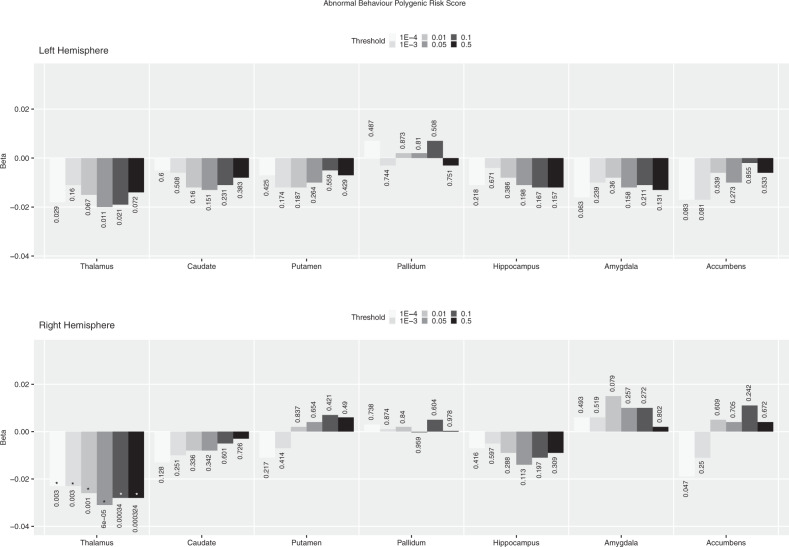
*Abnormal behaviour* gene set PRS calculated a different *p* thresholds association with left and right subcortical volumes. The direction and length of the bar correspond to the Beta value, the associated uncorrected *p* value is added for each Beta/bar. For comparison, this figure also includes the results for the *p*-threshold < 0.05 used in previous analyses. FDR correction based on 70 tests (FDR significant results are mark with an asterisk).

We further investigated the association between *abnormal behaviour PRS* and subcortical volumes. We found negative FDR-corrected significant association between this *PRS* at all *p*-thresholds and the volume of the right thalamus, the strongest at p-threshold <0.05 as reported above (Fig. 3). *Abnormal behaviour* PRS, though, did not show any other stable association across *p*-thresholds with subcortical volumes, at either nominal or FDR-corrected significant level. For completeness, the results from the same analyses for all gene-sets considered previously are presented in the Supplemental Fig. 1. None of these showed FDR-corrected significant associations with subcortical volumes, and only *abnormal nervous system electrophysiology PRS* and *FMRP targets PRS* showed stable negative nominal associations across several *p*-thresholds with the left caudate and the left thalamus respectively.

## Discussion

In this proof-of-concept study we revisited the association between polygenic risk for SZ and subcortical volumes using the largest and therefore most powered GWAS published to date^4^ in a uniquely large sample of unaffected participants, also examining the effect of restricting common allele risk to gene sets known to be enriched for SZ^4,21^. Our results showed that schizophrenia polygenic risk restricted to certain gene sets predicted more variance of neuroimaging biomarkers than an overall genomic approach. Moreover, we found considerable heterogeneity on the effects over subcortical volumes of different gene sets.

As previously reported using earlier GWAS training data^8–10^, we did not find any significant associations between *genomic PRS* for SZ and bilateral subcortical volumes. Dividing this into its *genic* and *intergenic* components did not dramatically change that picture, both these scores showing no significant associations with any subcortical volume, with the exception of nominal negative associations between *intergenic PRS* and putamen and amygdala volumes. Accounting for hemisphere changed these results only slightly, with nominal associations with left putamen and right pallidum now appearing, but none surviving FDR correction. Of notice, though, the hemispheric divide clearly strengthened the association between the *intergenic PRS* and amygdala volume, confined to the right hemisphere, despite still falling short of the FDR-corrected threshold. Interestingly, though, *genic PRS* showed not even a trend to associate with the volume of the amygdala. This result suggests that long-range gene regulatory variation lying outside our genic windows account for most of the volume variability within the right amygdala. If replicated, the importance of intergenic effects will bring the challenge of further dissecting core elements within it.

The analyses of *gene-set PRS* brought a different picture of the association between common allele risk for SZ and subcortical anatomy. The *abnormal behaviour PRS* showed a negative FDR-corrected significant association with the volume of the thalamus. Considering hemisphere, this association was present at FDR-corrected level for right thalamus, whereas only at nominal level for the left thalamus. The *LoF intolerant PRS* and the *FMRP targets PRS* also showed a negative nominal association with the size of the thalamus, with the latter also being associated with hippocampal (negative) and pallidum (positive) volumes. The strategy of limiting our PRS calculations to *gene-sets*, as opposed to a *genomic* or *genic* approach, proved to be more successful in capturing subcortical anatomical variation.

Since the selection of the SNP *p*-threshold initially used (i.e., *p* < 0.05) was based on its ability to best capture the global polygenic common variant risk burden for SZ, but not necessarily variants associated with brain anatomy, we repeated our analyses using a wide range of *p*-thresholds above and below the initial 0.05. Overall, results showed that *p* < 0.05 was not the optimal threshold in most instances. For example, the previous nominal association between *genomic PRS* and the volume of the right pallidum, reached FDR-corrected significance level with a PRS calculated at a *p*-threshold < 0.01. Similarly, the association between *intergenic PRS* and the volume of the pallidum and amygdala would have been FDR-significant if initially calculated at *p*-threshold < 0.01; however, since at this stage we were performing a larger number of tests, these fell short of the FDR threshold. In relation to the association between *genomic PRS* and volume of the pallidum, the latter has shown to be increased in participants with SZ compared to healthy participants^5,6^; however, we find that the higher the common allele risk for SZ, the smaller the volume of the pallidum. Previous research from our group has shown the same negative association in a much smaller sample of healthy participants using an earlier GWAS as the training dataset^31^, and also comparing healthy participants carrying rare variants penetrant for SZ against participants not carrying any damaging rare allele^15^. Together, these findings suggest that previous clinical research showing larger pallidum in people with SZ could have been concealed by confounders linked to clinical status—e.g., use of antipsychotic drugs^32^, suggesting that this would not be a trait marker of risk, but a state marker of the presence of the disorder.

*Abnormal behaviour PRS* showed FDR-corrected significant associations with the right thalamus volume for all *p*-thresholds applied, proving the stability and robustness of this association. Interestingly, the analyses using *genomic* and *genic PRS* did not detect any association with thalamic size, suggesting that only a restricted number of variants included in genes within this gene-set may determine the development of the thalamus to its adult size. The inclusion of many other variants in ‘omic’ approaches with potentially no-effect—or effects in the opposite direction—will dilute this association making it invisible to commonly used approaches. It is worth noting as well that this gene-set PRS did not show any clear pattern of association with any other subcortical volume, suggesting a rather specific effect of genes included in it, at least with regard to subcortical anatomy. The *abnormal behaviour* gene set consists of genes identified as modifying behaviour via single-gene manipulation studies in mouse. The range of behavioural tests, and by implication the range of neural circuits, covered by this set is extremely broad, which complicates any mechanistic interpretation of the associations found in our study. It seems likely that considerably larger imaging cohorts will be required to identify more specific functional associations within this set of genes that could be interpretable from a biologically mechanistic approach. The PRS restricted to the *FMRP targets* gene-set also showed a stable association with the volume of the left thalamus across several *p*-thresholds; however, these only reached nominal significance and would require further replication in more powered samples before any conclusions can be extracted. All these associations between common allele risk and thalamic volume followed the predicted direction based on previous clinical research^5,6^; that is, a negative association indicating that the higher the risk for the disorder, the lower the volume of the thalamus, making it a target for research into SZ risk biomarkers.

Other interesting results from our gene-set analysis are: (1) The positive nominal significant association between *FMRP targets PRS* and pallidum volume, opposing the negative association of this volume with *genomic PRS* discussed above. It could be argued that only variants within FMRP target genes influence the pallidum’s development in the direction of risk for SZ, most other variants potentially influencing in the opposite direction. However, due to this association not surviving FDR correction, this interpretation remains rather speculative and would require replication in more powered samples before any conclusions can be drawn. (2) Despite the required caution interpreting negative results, it is surprising that *LoF intolerant PRS* did not show any robust association with subcortical volumes, when considering that this gene set explains the largest amount of SNP heritability and shows the strongest association with SZ compared to other gene sets. However, it is important to note that gene sets that appear scarcely associated with subcortical volumes could exert their influence on SZ via other relevant biomarkers (e.g., cortical anatomy or white matter content) not explored here.

There are caveats to our work that require mentioning: (1) Although, to our knowledge, this is the largest dataset of healthy participants ever interrogated for association between common allele risk and subcortical volumes, statistical power is still limited when considering the small effect sizes obtained. Future releases of UK Biobank data and other large imaging/genetic resources should allow for replication studies and meta-analyses. (2) We based our PRS on the latest/largest GWAS published to date^4^, but the future release of the results from the Psychiatric Genomics Consortium’s latest GWAS should allow for a more accurate and powered calculation of PRS. Also, whilst our study did not include patients, there is still a small chance that a few of the healthy participants from UKBB were included as controls in Pardiñas et al. GWAS^4^; future studies should therefore try to de-duplicate these samples. (3) We selected gene sets based on previous work from our group showing enrichment for SZ variants^4,21^; however, this is not necessarily an optimal criterion when testing gene-set PRS association with brain phenotypes. In this respect, future work should develop methods to test optimal aggregation of variants that can best predict these—and other—phenotypes. (4) Finally, it is important to note that due to the recruitment strategy, the UK Biobank cohort is not a truly representative sample of the general population but a cohort of participants on average healthier and demographically more educated and wealthier^33^.

In summary, we present here the results of a proof-of-concept study in which we challenge the use of an ‘omic’ PRS approach to examine the effects of SZ allele risk on brain phenotypes. The alternative strategy of limiting PRS calculations to smaller gene-sets based on an a-priori biologically informed criterion proved more successful in capturing subcortical volume variance than the genomic or genic approach, supporting the idea that common allele risk for SZ impact subcortical brain anatomy and suggesting thalamic volume as an important target in future research into risk markers for SZ. The observed level of heterogeneity across gene-set PRS effects on subcortical volumes could partly explain the lack of success in previously published genome-wide PRS approaches. Future research should focus on the optimisation of gene-sets that could better account for interindividual brain variability, potentially inputting information on gene expression at key developmental stages and its potential application for patient stratification based in this neurobiologically driven strategy.

## Supplementary information

Supplementary Fig. 1
