# Supplementary material for: Polygenic risk for schizophrenia and subcortical brain anatomy in the UK Biobank cohort
Source: Transl Psychiatry. 2020 Sep 9;10:309. doi: 10.1038/s41398-020-00940-0 (PMC7481214; doi:10.1038/s41398-020-00940-0)
Supplement: Supplementary file 1 — Supplementary Fig. 1 [file 41398_2020_940_MOESM1_ESM.docx]

**Supplemental Figure 1.** Gene set PRS other than *abnormal behaviour* PRS calculated a different p thresholds association with left and right subcortical volumes. The direction and length of the bar correspond to the Beta value, the associated uncorrected p value is added for each Beta/bar. For comparison, this figure also includes the results for the p-threshold <0.05 used in previous analyses. FDR correction calculated independently for each PRS and based on 70 tests in each case.
